# Mechanical Stabilization of Alginate Hydrogel Fiber and 3D Constructs by Mussel-Inspired Catechol Modification

**DOI:** 10.3390/polym13060892

**Published:** 2021-03-14

**Authors:** Kyoungryong Kim, Jae Hyuk Choi, Mikyung Shin

**Affiliations:** 1 Department of Biomedical Engineering, Sungkyunkwan University (SKKU), Seobu-ro 2066, Jangan-gu, Suwon 16419, Gyeonggi-do, Korea; ryong9038@naver.com; 2 Department of Intelligent Precision Healthcare Convergence, Sungkyunkwan University (SKKU), Seobu-ro 2066, Jangan-gu, Suwon 16419, Gyeonggi-do, Korea; wre56@naver.com

**Keywords:** alginate, catechol, wet-spinning, 3D structure, inter-catechol crosslinking

## Abstract

Alginate is a representative biocompatible natural polymer with low cost for a variety of biomedical applications, such as wound dressing, drug delivery systems, tissue scaffolds, and 3D bioprinting. Particularly, the rapid and facile gelation of alginate via ionic interactions with divalent cations has been used for in situ 3D hydrogel fiber formation, which is potentially applicable to engineering cell alignment. However, challenges in enhancing the mechanical properties of alginate hydrogel fibers under physiological conditions are unresolved because of their fast dissociation by ion exchange. Herein, we report a stabilization strategy for alginate hydrogel fibers through mussel-inspired catechol chemistry, which involves inter-catechol crosslinking within a few minutes under basic conditions. The fabrication of catechol-tethered alginate hydrogel fibers through wet-spinning enabled the design of mechanically strong 3D constructs consisting of fibers. Catechol-to-quinone oxidation followed by covalent crosslinking enhanced the tensile strength of a single fiber. Additionally, the ‘gluing’ capability of the catechol stabilized the interface among the fibers, thus retaining the shape fidelity of the 3D constructs and encapsulating the cell density during culture. Our findings will be useful for designing bioink materials specialized in fibrous-type tissue scaffolds with mechanical stability.

## 1. Introduction

Alginate is a linear polysaccharide consisting of (1-4)-linked β-D-mannuronate and α-L-guluronate extracted from brown algae. Alginate is a representative material that has great biocompatibility and low cost [1,2]. Moreover, alginate forms a hydrogel by ionic crosslinking with divalent cations, such as Ca^2+^, Ba^2+^, and Cu^2+^ [3]. The gelation of alginate can be utilized for wound dressing, drug and macromolecule (e.g., protein) encapsulation, and cell transplantation [1,2,3]. Additionally, the gelling property of alginate offers the advantage of building scaffolds. Alginate can form fibers via wet-spinning process and be used as a bioink for 3D bioprinting [4,5,6,7,8,9,10,11,12,13]. Typically, Ca^2+^ ions are used for alginate gelation, and alginate gel formation is promptly proceeded so alginate fiber can be formed by just injecting the alginate solution into a CaCl_2_ solution [4,5,6,7]. 3D bioprinting usually constructs a 3D structure in fiber units; thus, alginate fiber formation methods can be applied for 3D bioprinting [8,9,10,11,12,13]. However, alginate gel is rapidly degraded under physiological conditions because of the elimination of ionic bonds between the alginate polymer chains and divalent cations by ion exchange [14]. This makes it difficult to use alginate alone and limits wide application of alginate. To overcome this limitation, functionalized alginates, such as poly(ethylene glycol)-diamine conjugated alginate and methacrylate conjugated alginate, have been reported [1,2,15,16]. These alginate hydrogels have covalent crosslinks and thus exhibit high mechanical stability.

Catechol-functionalized alginate (Alg-Ca) is one such compound [17,18]. Catechol is a mussel-inspired adhesive functional group capable of exhibiting various interactions such as catechol-metal chelation, electrostatic interaction, π−π stacking, and hydrogen bonds [19]. Additionally, catechol is rapidly oxidized to quinone under basic condition. Quinone is a highly reactive functional group, and catechol functionalized polymers can be crosslinked by forming inter-catechol covalent bonds through the quinone form [19,20]. For these reasons, catechol modification can be applied to various polymers, such as poly(ethylene glycol), hyaluronic acid, and gelatin [21,22,23]. These catechol interactions can also be used to enhance the mechanical stability of bulk alginate hydrogels under physiological conditions and achieve sustained drug release from the hydrogels [17,24]. However, most studies have focused on the effect of catechol in 3D bulk hydrogels, rather than small fine structures (e.g., fibers) that have dimensions of the order of several millimeters.

**Figure 1:**
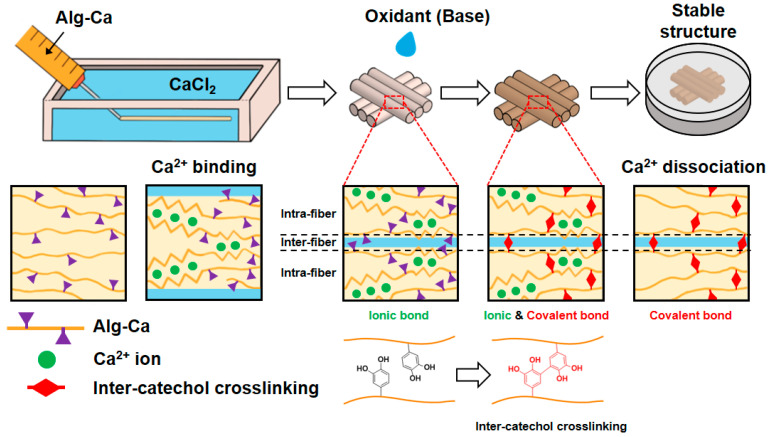
Schematic illustration of preparation of wet-spun Alg-Ca fibers and their 3D fibrous constructs with oxidative treatment. The fibers and their 3D structure are fabricated using initial ionic bonds followed by inter-catechol crosslinking even after dissociation of ionic bonds.

In this study, we prepared an Alg-Ca hydrogel fiber via wet-spinning (Figure 1). The short-term exposure of the hydrogel fiber to basic conditions (e.g., <5 min) enabled the formation of inter-catechol covalent bonds inside a single fiber as well as among the fibers, thus increasing the mechanical stability of the Alg-Ca fiber under physiological condition even after the fabrication of the 3D constructs. Additionally, the mechanically robust Alg-Ca fibers after base treatment could maintain a high cell density for a single day culture compared to that of the unoxidized fibers. Our findings regarding the catechol chemistry to increase the mechanical stability of hydrogel fibers is promising for engineering a new type of polymeric ink for further 3D bioprinting techniques.

## 2. Materials and Methods

### 2.1. Materials

Alginic acid sodium salt from brown algae (medium viscosity), dopamine hydrochloride, N-hydroxysuccinimide (NHS), 2-(N-morpholino)ethanesulfonic acid (MES) solution (1 M), Dulbecco’s phosphate buffered saline (DPBS) with calcium chloride and magnesium chloride, Trizma base, Trizma hydrochloride, and N-(2-Hydroxyethyl)piperazine-N′-(2-ethanesulfonic acid) (HEPES) buffer solution (1 M) were purchased from Sigma Aldrich (St. Louis, MO, USA). 1-(3-Dimethylaminopropyl)-3-ethylcarbodiimide hydrochloride (EDC-HCl) was purchased from Tokyo Chemical Industry (Tokyo, Japan). Phosphate buffered saline (PBS, 10X, pH 7.2) was purchased from WELGENE (Gyeongsan, Korea). Anhydrous ethyl alcohol was purchased from Daejung (Siheung, Korea). Fetal bovine serum (FBS, qualified), penicillin-streptomycin and Dulbecco modified eagle medium (DMEM) were purchased from Gibco (Waltham, MA, USA). LIVE/DEAD viability/cytotoxicity kit for mammalian cells was purchased from Invitrogen (Waltham, MA, USA). SYLGARD 184 silicone elastomer base and SYLGARD 184 silicone elastomer curing agent were purchased from Dow (Midland, MI, USA). SpectraPor 1 Dialysis Membrane (Standard RC Tubing, molecular weight cut off (MWCO) = 6–8 kDa) was purchased from Spectrum (Rancho Dominguez, CA, USA).

### 2.2. Synthesis and Characterization of Alg-Ca

To synthesize Alg-Ca, EDC/NHS coupling reaction was used. Alginate (Alg, 1 g, almost 5 mmol as the number of Alg repeating units) was dissolved in 100 mL of MES buffer (0.1 M, pH 5.6). Sequentially, EDC-HCl (5 mmol) and NHS (5 mmol) were added. After the addition, the solution was stirred for 20 min in a nitrogen atmosphere. Dopamine hydrochloride (5 mmol, 1 equivalent to the number of carboxyl groups of Alg) was dissolved in 3 mL of MES buffer (0.1 M, pH 5.6). Subsequently, the dopamine hydrochloride solution was injected into the reaction solution dropwise using a syringe. The reaction was continued for 1 h at room temperature (25 °C). After the reaction, the solution was dialyzed (using a 6–8 kDa MWCO membrane) in 5 L acidified deionized water (DW) (pH 6) with 20 g of NaCl for 3 days. After 3 days, the solution was dialyzed in DW for 4 h. The solution was then lyophilized 7 days. The degree of substitution was evaluated by ^1^H NMR spectroscopy using Varian Oxford 300 MHz NMR (Varian, Palo Alto, CA, USA) and absorbance at 280 nm (A_280_) measured by an Agilent 8453 UV–vis spectrometer (Agilent Technologies, Santa Clara, CA, USA). Dopamine hydrochloride (5–50 μg/mL) was used to create a standard curve of A_280_ depending on the concentration of the catechol moiety.

### 2.3. Rheology

A Discovery Hybrid Rheometer 2 (TA instrument, New Castle, DE, USA) with a 20 mm parallel plate geometry was used for the rheological test. The shear viscosity of the Alg and Alg-Ca solutions (3 *w*/*v* %) in the DW and HEPES buffer (25 mM, pH 7.4) as a function of the shear rate (0.01–1000 s^−1^) was evaluated at room temperature (Gap = 200 μm). To quantify the shear-thinning behavior of Alg and Alg-Ca, power-law model was applied as the equation

Shear viscosity = Κ × (Shear rate)^n − 1^, (1)

where Κ is the consistency index and show shear viscosity at low shear rate; n is power-law index [25]. For shear-thinning behavior, n is lower than 1.

### 2.4. Wet-Spinning of Alg and Alg-Ca Fibers

A calcium chloride solution (2 *w*/*v* %) was prepared in DW, 10% (*v*/*v*) ethanol, and 20% (*v*/*v*) ethanol as the coagulation solution. To fabricate the fiber, the Alg and Alg-Ca solutions (3 *w*/*v* % in DW) were injected into coagulation bath using an 18-gauge (18 G) blunt needle at a speed of 0.6 mL/min. The initial diameter of the fibers was measured using a Nikon Eclipse Ts2 optical microscope with a Nikon DS-Fi3 microscope camera (Nikon, Tokyo, Japan) and ImageJ software. The wet-spinning of the Alg-Ca fiber using 27-gauge (27 G) blunt needle using CaCl_2_ solution (2 *w*/*v* %, in 20 *v*/*v* % ethanol) was also tested, and the initial diameter of the fibers was measured.

### 2.5. Swelling Kinetics and Tensile Test of Alg and Alg-Ca Fibers in Physiological Condition

The wet-spinning of Alg and Alg-Ca (3 *w*/*v* % in DW) was performed using a CaCl_2_ solution (2 *w*/*v* %, in 20 *v*/*v* % ethanol) and an 18 G blunt needle. The fibers were soaked in CaCl_2_ solution for 10 min to ensure complete crosslinking. Subsequently, the Alg and Alg-Ca fibers were soaked in DW, Tris buffer (50 mM, pH 8.5), respectively, for 20 min. The swollen fibers were cut into 2.5 cm pieces and dried for a day at room temperature. The terminal of the dehydrated fiber pieces was fixed on a polydimethylsiloxane (PDMS) mold (1 × 1 cm^2^). The fixed fiber pieces were used to demonstrate the swelling kinetics, tensile stress, tensile strain, and the Young’s modulus of the fiber in PBS (1X, pH 7.4). The expansion ratio was used as an indicator of the swelling kinetics and was calculated as

Expansion ratio (%) = (Diameter of fiber/Diameter of dehydrated fiber − 1) × 100(2)

Optical microscopy and ImageJ software were used to measure the diameters of the fibers (digital vernier calipers were used to measure the diameter of the large fibers). After measuring the diameter of the dehydrated fibers, the fibers were swollen in PBS (1X, pH 7.4), and their diameters were measured at predetermined times (5, 10, and 30 min, 1, 2, 4, 8, 24, 48, 72, and 96 h). Additionally, fixed dehydrated fiber pieces were used for the tensile tests. The fibers were swollen in PBS (1X, pH 7.4) for 1 h and the tensile test was performed after removing the solution on the surface of the fiber. The tensile tests were conducted using an INSTRON 5583 universal testing machine (INSTRON, Norfolk, MA, USA) with a 50 N load cell and INSTRON Bluehill software at a speed of 20 mm/min. The stress was calculated using the average fiber diameter at 1 h obtained from the swelling kinetics, assuming that the cross-section of the fiber was circular, and using the load data from the tensile test.

### 2.6. Three-Dimensional Construction Using Wetspun Fibers

The fibers were prepared using the Alg and Alg-Ca solution (3 *w*/*v* % in DW), CaCl_2_ solution (2 *w*/*v* % in 20 *v*/*v* % ethanol), and an 18 G blunt needle at a speed of 0.6 mL/min. Subsequently, the fibers were manually stacked to construct 3D structure. A minimal volume (droplet) of 0.1 M NaOH was dropped to the stacked fibers and incubated at 37 °C for varying time durations (3, 5, and 10 min). Subsequently, the constructed 3D structures were soaked in PBS (1X, pH 7.4, 25 °C). After 24 h, the state of 3D structures, which was used to determine the incubation time of subsequent NaOH treatment processes, was confirmed.

### 2.7. Cell Viability and Density

L929 cells were used for in vitro studies and cultured in DMEM with 10% (*v*/*v*) FBS and 1% (*v*/*v*) penicillin-streptomycin. Subculture was performed every 3 days. The cell suspension was prepared at 4 × 10^6^ cells/mL in DMEM with 1% penicillin-streptomycin. The cell suspension was mixed with the Alg or Alg-Ca solution (4 *w*/*v* %) dissolved in the HEPES buffer (25 mM, pH 7.4) at a ratio of 1:3 (final concentration of the cell-loaded solution = 3 *w*/*v* %, final cell density of the solution = 1 × 10^6^ cells/mL). Subsequently, the cell-loaded solution was used for wet-spinning with the CaCl_2_ solution (2 *w*/*v* %, in 20 *v*/*v* % ethanol) and an 18 G blunt needle. After fabricating the fibers, 0.1 M NaOH was used to treat the fibers for 5 min at 37 °C, and the fibers were washed in pre-warmed DPBS to prevent the contamination of media (the not-treated (NT) group was washed in DPBS without NaOH treatment and incubation).

After washing, the fibers were transferred to a 24 well plate with media similar to the culture conditions. After 1 day, the cells that existed in the fiber were stained using a LIVE/DEAD viability/cytotoxicity kit. The cell viability and density were evaluated using Leica TCS SP8 STED confocal microscopes (Leica, Wetzlar, Germany, z-step size = 20 μm) with a 20 Ø confocal dish (SPL Life Science, Pocheon, Korea) and ImageJ software with the ClearVolume plugin.

### 2.8. Statistical Analysis

The statistical significance was evaluated using the one-way ANOVA test with a post hoc Tukey test.

## 3. Results and Discussion

### 3.1. Characterization of Alg-Ca

**Figure 2:**
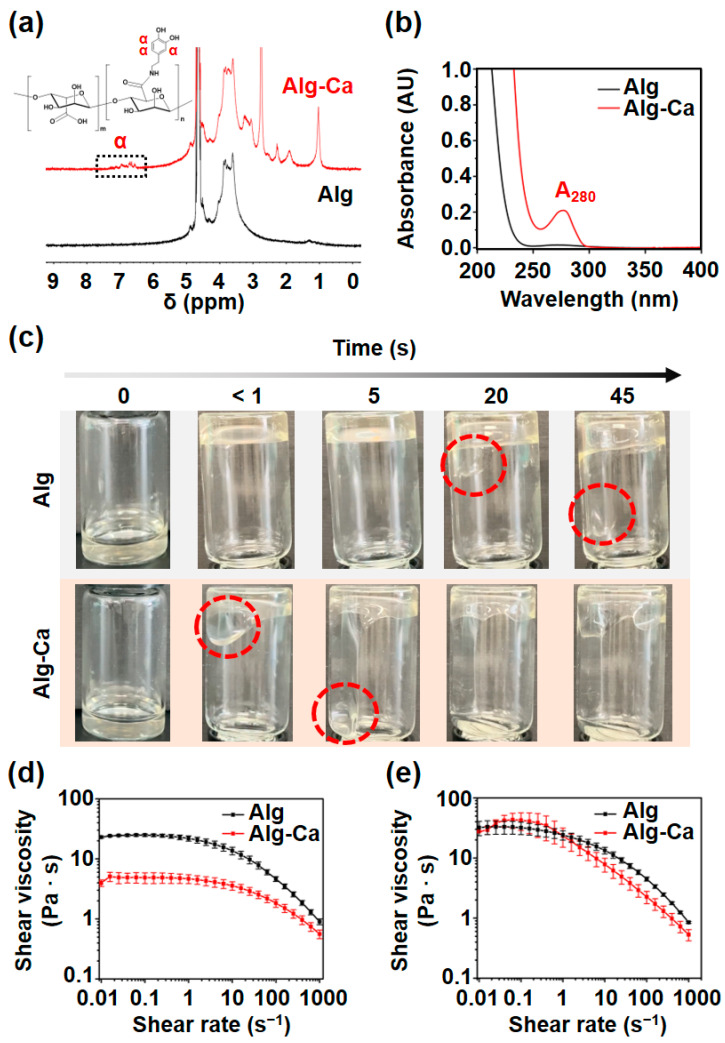
Physicochemical characteristics of Alg-Ca compared to Alg. (**a**) ^1^H NMR spectrum of Alg (black) and Alg-Ca (red). Alg-Ca ^1^H NMR spectrum exhibited proton peaks that indicate the appearance of the catechol α proton. (**b**) UV–vis spectrum of Alg (black) and Alg-Ca (red) aqueous solution. (**c**) Viscosity comparison of Alg (top) and Alg-Ca (bottom) solution (3 *w*/*v* % in DW) in 10 mL vial. Red dashed lines indicate the solution flowing down. Shear viscosity of Alg (black) and Alg-Ca (red) in DW (**d**), and in HEPES buffer (25 mM, pH 7.4) (**e**) depending on shear rate (0.01–1000 s^−1^) (n = 3, mean ± SD).

Prior to the fabrication of the hydrogel fibers, Alg-Ca was synthesized via typical EDC/NHS coupling reaction. During the reaction, the amine group of dopamine was conjugated to the carboxyl group of Alg. In Figure 2a, the ^1^H NMR spectrum of Alg-Ca exhibited proton peaks from 7.12 to 6.57 ppm (‘α’ peak), which indicate that the appearance of the proton exists on aromatic ring of the catechol group. Furthermore, UV–vis spectrum showed the absorption peak of Alg-Ca at a wavelength of 280 nm because of the aromatic ring of the catechol group; in contrast, the peak was not observed in the unmodified Alg (Figure 2b). The degree of substitution was calculated as 1.59% by A_280_ (= 0.2). Although the further study might be required for synthesize optimization to control the degree of catechol substitution, the 1.59% substitution was sufficient to compare Alg-Ca to Alg in the following wet-spinning process.

### 3.2. Shear Viscosity of Alg-Ca

While preparing the Alg and Alg-Ca solutions (3 *w*/*v* % in DW), the Alg-Ca solution seemed to have a lower viscosity than the Alg solution. Low viscosity can be helpful in preparing and handling solutions and simplifying the manufacturing process. Additionally, shear viscosity is a crucial factor in bioprinting. The viscosities of the Alg and Alg-Ca solutions (3 *w*/*v* % in DW) in 10 mL vials were then compared, and their shear viscosities at room temperature was evaluated using a rheometer. In the 10 mL vial, the Alg-Ca solution started to flow down immediately after the vial was turned over and the reached opposite end of the vial within 5 s (Figure 2c). In contrast, the Alg solution started to flow down after 20 s and reached the opposite end of the vial within 45 s (Figure 2c). These results show that the Alg-Ca solution has a lower viscosity than the Alg solution. When dissolved in DW (3 *w*/*v* %), both the Alg and Alg-Ca solution exhibited shear-thinning properties and the Alg-Ca solution exhibited lower shear viscosity than the Alg solution over the entire range (Figure 2d). The Κ value of Alg-Ca was also much lower than Alg (Table 1). Alg-Ca can be applied in cases where low viscosity is required (e.g., bioprinting). The low viscosity might be due to partial folding effect of alginate chain by non-covalent intramolecular interactions between catechol and the polymer (e.g., multiple hydrogen bonds), decreasing relative molecular weight of the polymer [26].

The shear viscosity in the HEPES buffer (25 mM, pH 7.4) was also evaluated for the solution prepared with cells (Figure 2e). HEPES buffer was used instead of PBS to avoid the aggregation of the Alg polymer chains. The Κ value of Alg in the HEPES buffer did not show a statistically significant difference from the Κ value of Alg in DW (Table 1). This indicates that the HEPES buffer is useful to avoid the aggregation of Alg. However, the Κ value of Alg-Ca in the HEPES buffer was similar to the Κ value of Alg in HEPES buffer and higher than the shear viscosity of Alg-Ca in the DW (Table 1). This may have been caused by the interaction between the HEPES molecule and catechol group in the Alg polymer chain. HEPES is a zwitterion in the biological pH range, and some of the nitrogen in the HEPES molecule exists in the quaternary ammonium form [27]. The catechol group has strong cation–π interactions with quaternary ammonium [20], which may cause an increase in the shear viscosity of the Alg-Ca solution. Therefore, the viscosity of Alg-Ca can be different depending on the solvent; using buffers other than HEPES and PBS when preparing the Alg-Ca solution with cells, may be necessary to take advantage of the lower shear viscosity of Alg-Ca compared to Alg. In the HEPES buffer, both Alg and Alg-Ca exhibited shear-thinning properties.

### 3.3. Stabilization of Alg-Ca Wet-Spinning

**Figure 3:**
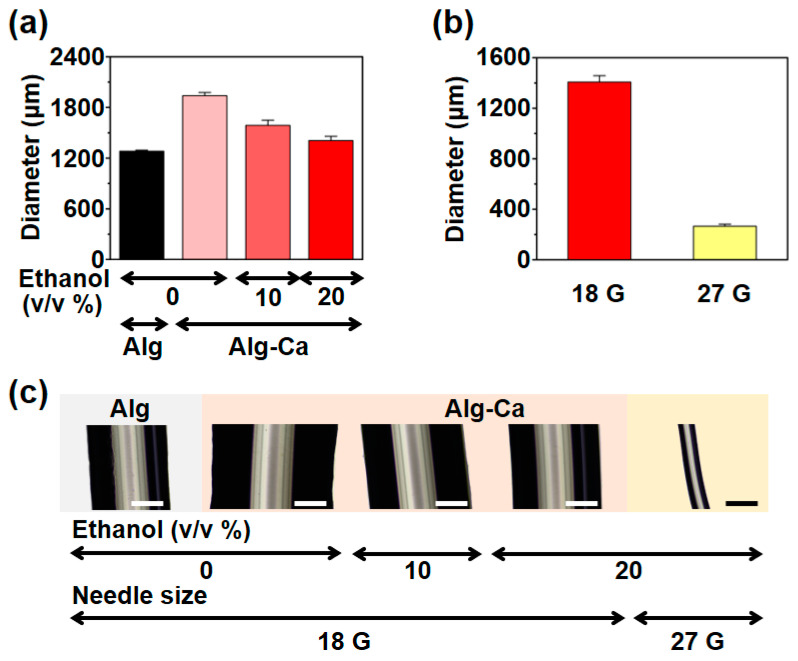
Initial diameter of Alg-Ca fibers depending on wet-spinning condition. (**a**) Initial diameter of Alg fiber (first bar) and Alg-Ca fiber (second–fourth bar) depending on ethanol concentration of CaCl_2_ solution (*n* = 3, mean ± SD). (**b**) Initial diameter of Alg-Ca fiber fabricated with 20% (*v*/*v*) ethanol CaCl_2_ solution using 18 G (red) and 27 G (yellow) blunt needle (*n* = 3, mean ± SD). (**c**) Representative images of Alg (first) and Alg-Ca (second—fifth) fibers (scale bar = 500 μm).

Before fabricating the Alg-Ca fiber, the gelling rate and capacity of Alg-Ca were expected to be lower than those of Alg, because parts of the carboxyl group in the Alg polymer chain, which is a crucial part of the ionic bond between the Alg polymer chain and divalent cation, were consumed to conjugate the catechol group; the chelation of the catechol group may have been affected. The catechol group has a high affinity for metals [28]; thus, the catechol group may chelate divalent cations and hinder divalent cation binding to the Alg polymer chain. Therefore, the wet-spinning of Alg-Ca had to be tested to evaluate the gelling rate and capacity of Alg-Ca to form the fiber. Initially, the Alg-Ca fiber was fabricated with 2% (*w*/*v*) CaCl_2_ aqueous solution for testing; however, the Alg-Ca fiber was significantly larger than the Alg fiber fabricated under similar conditions. Additionally, Alg-Ca frequently formed bulk hydrogels instead of fibers. This indicates that the gelling rate of Alg-Ca is considerably lower than the gelling rate of Alg. Ethanol was added to the CaCl_2_ solution to stabilize the Alg-Ca wet-spinning process. Ethanol is a non-solvent of Alg and in wet-spinning, non-solvents are often used to stabilize the coagulation process [24,29]. The size of the Alg-Ca fiber depending on the ethanol concentration was evaluated, and the size of the fiber was determined using the diameter of the fiber. The Alg fiber fabricated using 2% aqueous CaCl_2_ solution had a diameter of 1281 ± 13 μm, whereas the Alg-Ca fiber fabricated using the same solution had a diameter of 1939 ± 37 μm (Figure 3a,c). After using ethanol, the Alg-Ca fiber exhibited a lower diameter. The Alg-Ca fiber fabricated using 2% CaCl_2_ in 10% (*v*/*v*) ethanol had a diameter of 1587 ± 63 μm, and 1407 ± 51 μm in 20% (*v*/*v*) ethanol (Figure 3a,c). Similarly, the Alg-Ca fiber could be fabricated at the level of a typical Alg fiber using 20% (*v*/*v*) ethanol as a solvent of CaCl_2_; 20% (*v*/*v*) ethanol was used in all subsequent wet-spinning processes. The fabrication of the Alg-Ca fiber using a 27 G blunt needle was also demonstrated for further application, and the diameter was 265 ± 16 μm (Figure 3b,c).

### 3.4. Stable Alg-Ca Fiber in Physiological Condition

**Figure 4:**
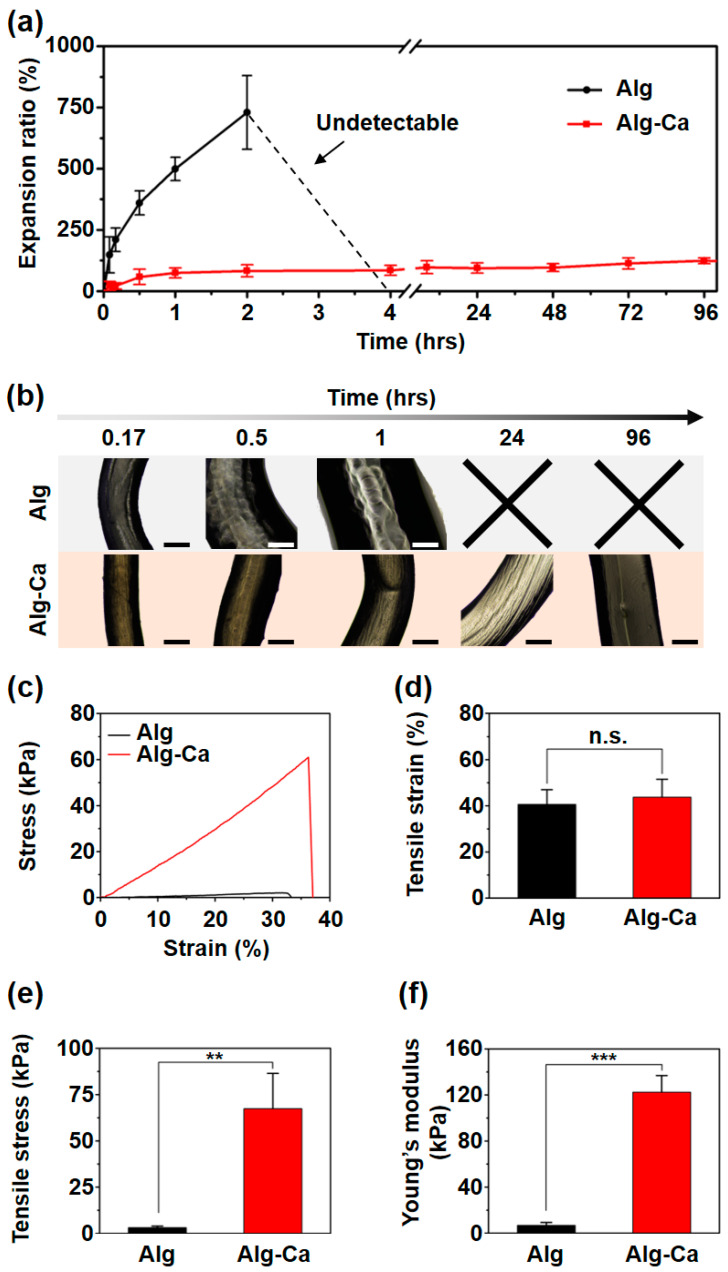
Comparative observation of Alg and Alg-Ca fiber swollen in PBS (1X, pH 7.4). (**a**) Swelling kinetics of Alg (black) and Alg-Ca (red) fiber (*n* = 3, mean ± SD). Alg fiber pieces were fractured and dissociated between 2 and 4 h (black dotted line) and the diameter of the fiber could not be determined. (**b**) Representative images of Alg (top) and Alg-Ca (bottom) fiber (scale bar = 500 μm). Representative stress–strain curve (**c**), tensile strain (**d**), tensile stress (**e**), and Young’s modulus (**f**) of Alg (black) and Alg-Ca (red) fiber after 1 h of swelling (*n* = 3, mean ± SD) (** *p* < 0.01, *** *p* < 0.001, n.s. = not significant).

After stabilizing the Alg-Ca wet-spinning and fabricating the Alg-Ca fiber, the swelling kinetics in the PBS (1X, pH 7.4) and tensile tests of the Alg-Ca fibers were evaluated to demonstrate the stability of the Alg-Ca fibers under physiological conditions. The fabricated Alg-Ca fibers were soaked in a Tris buffer (50 mM, pH 8.5) to induce the oxidation of the catechol group on the Alg polymer chain. The catechol group was oxidized to catecholquinone and catecholquinone crosslinked Alg polymer chains via inter-catechol covalent bonds [17,19]. A drying process was required to fix the fiber to measure the diameter at the same position of the fibers and enable tensile test. In the PBS, the Alg fibers expanded significantly more than the Alg-Ca fibers and were fractured and dissociated between 2 and 4 h (Figure 4a,b). Ion exchange occurred in the PBS, and the bond between the Alg and Ca^2+^ was removed. Therefore, it can be inferred that the Alg fibers cannot maintain their fiber structure. However, the Alg-Ca fibers reached swelling equilibrium after approximately 2 h and maintained their fiber structure for over 96 h (Figure 4a,b). The inter-catechol covalent bonds in the Alg-Ca fiber were maintained in the PBS and maintained the structure of Alg-Ca fiber even when the Ca^2+^ ions were released. Tensile tests were conducted using the fibers after swelling for 1 h in the PBS. The stress–strain curve showed that the Alg-Ca fibers have considerably stronger mechanical properties than the Alg fibers (Figure 4c). The tensile strain of the Alg (41 ± 6%) and Alg-Ca (44 ± 8%) fibers did not exhibit a significant difference (Figure 4d). The tensile stress of the Alg fiber was 3 ± 1 kPa, and that of Alg-Ca fiber was 67 ± 19 kPa (Figure 4e). The Alg-Ca fiber had a tensile stress almost 22 times that of Alg fiber. The Young’s modulus of the Alg fiber was 7 ± 3 kPa and 122 ± 15 kPa for the Alg-Ca fiber (Figure 4f). The Young’s modulus of the Alg-Ca fiber was almost 18 times that of the Alg fiber. After swelling for 1 h in the PBS, most of ionic bonds between Ca^2+^ ions and either Alg or Alg-Ca in both fibers are reversibly dissociated. However, inter-catechol covalent bonds which are irreversible permit stable fiber shape fidelity of the Alg-Ca, indicating much higher tensile stress and the Young’s modulus than those of unmodified Alg. For similar tensile strain of Alg and Alg-Ca hydrogel fibers, tensile strain might depend on the type of the polymeric backbone and existence of non-covalent and reversible bonds involved in the crosslinking. Although the Alg-Ca might possess a few ionic bonds with calcium ions, the ionic bonds could not dramatically increase their tensile strain.

That is, the dominant factor for tensile strain might be the type of the polymeric chain, showing no significant difference in tensile strain of both fibers. The swelling kinetics and tensile tests demonstrated that the Alg-Ca fiber can maintain its fiber structure and has stronger mechanical properties than the Alg fiber under physiological conditions via inter-catechol covalent bonds.

### 3.5. Stable 3D Structure in Physiological Condition

**Figure 5:**
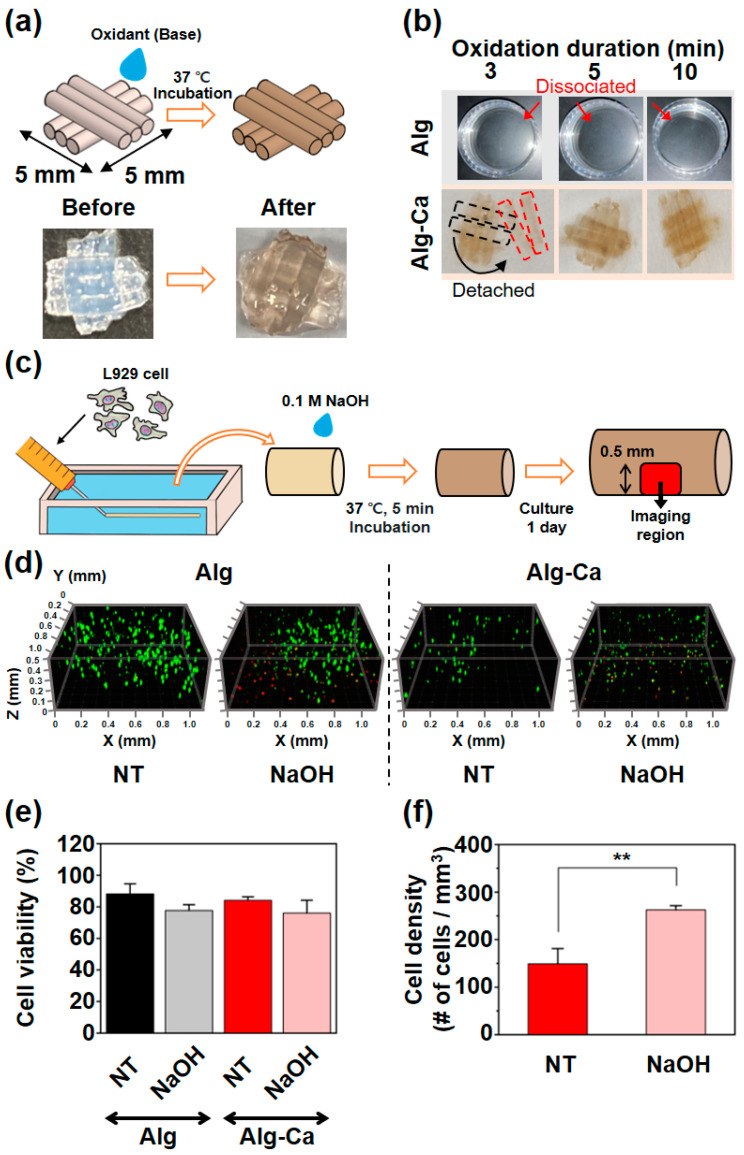
Constructing fiber-based 3D structure and fabricating cell loaded Alg-Ca fibers. (**a**) Schematic illustration of 3D structure and oxidant treatment (top), and images of 3D structure before (left bottom) and after (right bottom) treatment. (**b**) Images of 3D structure based on Alg (top) and Alg-Ca (bottom) fibers depending on oxidation duration after 24 h swelling in PBS. Alg fibers were dissociated (red arrow) and nothing remained in PBS. Black dashed line indicates original position of detached fiber (red dashed line). (**c**) Schematic illustration of procedure for fabricating cell loaded fibers and confocal imaged region (red square, depth = 0.5 mm). Result of live/dead cell staining, 3D view of Alg (left) and Alg-Ca (right) fibers (**d**), cell viability (**e**), and cell density (**f**). (**d**) Green for live cells and red for dead cells. Z = 0 is edge of fibers. (**e**) Cell viability of after a day culture in Alg fiber NT group (first bar), NaOH treated group (second bar) and Alg-Ca fiber NT group (third bar), NaOH treated group (fourth bar) (*n* = 3, mean ± SD). (**f**) Cell density of after a day culture in Alg-Ca fiber NT group (first bar) and NaOH treated group (second bar) (*n* = 3, mean ± SD) (** *p* < 0.01).

The stability of the fiber-based 3D structure via the inter-catechol covalent bond was also evaluated. First, to determine the oxidation duration, the 3D structure was manually constructed using wet-spun fibers (Figure 5a). In this process, NaOH (0.1 M) was used as the oxidant, but other substances that can induce oxidation (e.g., H_2_O_2_ with horseradish peroxidase) can be used to form inter-catechol covalent crosslinking [30]. A large amount of solution disassembles the structure or detaches the contact region between the fibers; therefore, a minimal volume of the NaOH was used. During incubation, the catechol group was oxidized, and inter-catechol covalent bonds were formed in both the intra-fiber region and inter-fiber regions. 3D structures constructed using the Alg fibers were fully dissociated, and only the solution remained (Figure 5b). However, 3D structure constructed using the Alg-Ca fibers retained not only single fibers but also entire 3D structures (Figure 5b). 3D structure constructed using the Alg-Ca fibers and incubated for 3 min also maintained a single fiber; however, some fiber pieces were detached; thus, the oxidation duration was determined as 5 min (Figure 5b). Treatment using the Tris buffer (50 mM, pH 8.5) instead of NaOH was also evaluated. However, it required a long oxidation period (over 30 min); thus, it was not suitable for biological use. These results showed that 3D structures constructed using Alg-Ca fibers were stable under physiological conditions through inter-fiber inter-catechol covalent bonds.

### 3.6. Cytotoxicity and Effectiveness of NaOH Treatment

One of the advantages of Alg wet-spinning is that it can encapsulate cells on inside the fibers. This is effective for cell transplantation and alignment and can also be used as a bioprinting model. To evaluate the potential of Alg-Ca for these applications, the cytotoxicity was evaluated. Confocal imaging was conducted at the center of the fiber (Figure 5c, depth = 0.5 mm). The 3D views showed that the cells located at the edge of the fiber were almost dead, but those cells that located at the center were almost alive in both the Alg and Alg-Ca fibers (Figure 5d). The cell viability values (the number of live cells/the number of cells) of the Alg fiber NT group, Alg fiber NaOH treated group, Alg-Ca fiber NT group, and Alg-Ca NaOH treated group were 88 ± 6%, 78 ± 4%, 84 ± 2%, and 76.0 ± 8%, respectively (Figure 5e). The cell density was calculated to evaluate the effectiveness of the NaOH treatment. A high cell density indicates a low fiber size and stable fiber. The cell density of the Alg-Ca fiber NT group was 149 ± 32 number of cells/mm^3^, and that of the Alg-Ca fiber NaOH treated group was 262 ± 9 number of cells/mm^3^ (Figure 5f). The NaOH treated group exhibited statistically significant enhancement of the cell density, indicating that the NaOH treatment significantly enhanced the structural stability of the Alg-Ca fiber. However, the Alg-Ca fibers with cells did not retain their fiber structure at the level of those without cells (using Alg-Ca dissolved in DW). These results of cell viability and density were considered to be a result of the movement of the NaOH; during the NaOH treatment, NaOH did not reach the center of the fiber and only affected the edge of the fiber, hence the cells at the center of the fiber were almost alive. However, NaOH did not reach the center of the fiber, which may have affected the oxidation level of the catechol group at the center of the fiber. Therefore, the structural stability of the Alg-Ca fiber under physiological conditions might not be sufficient. To achieve better structural stability and cell viability, a higher degree of substitution is required, and further studies that find proper buffer and oxidation conditions for fabricating cell-loaded fibers are required.

Although Alg-Ca needs improvement, the result of live/dead cell assay exhibited that Alg-Ca can be used instead of Alg to fabricate cell-encapsulated fibers with negligible cytotoxicity. Additionally, Alg-Ca maintained their fiber structure and the encapsulation density of the live cells even after NaOH treatment. Therefore, Alg-Ca has the potential as a bioink material specialized in fibrous-type tissue scaffolds with mechanical stability.

## 4. Conclusions

In this study, we demonstrated the structural stability of Alg-Ca fibers fabricated by wet-spinning and Alg-Ca based 3D structures under physiological conditions. Despite the loss of ionic bonds between the Alg polymer chains and Ca^2+^ ions, the Alg-Ca fibers retained their fiber structure by inter-catechol covalent crosslinking, which enhanced the stability of 3D structures by forming inter-catechol covalent bonds between the units of the 3D structure. This strategy can be applied to enhance the structural stability of other materials and the use of Alg alone in biomedical applications. Additionally, the potential of Alg-Ca as a bioink was demonstrated by evaluating the cytotoxicity of the Alg-Ca oxidation treatment. Many studies are still required for the application of Alg-Ca as bioink. However, we successfully demonstrated that our strategy can be applied in the design of a bioink material specialized in fibrous-type tissue scaffolds with mechanical stability.

## Figures and Tables

**Table 1:** Calculated value of Κ and n from power-law model.

Solvent	Sample	Κ	n
DW	Alg	16.6	0.87
	Alg-Ca	3.7	0.90
HEPES	Alg	19.3	0.84
	Alg-Ca	19.7	0.81

## Data Availability

The data presented in this study are available in article.
